# Controllable Dynamic Mechanical Cell Stimulation using Magnetically Actuated Artificial Cilia

**DOI:** 10.1002/adhm.202600001

**Published:** 2026-02-01

**Authors:** Roel Kooi, Tanveer Ul Islam, Oscar M.J.A. Stassen, Naomie Amsing, Jan de Boer, Jaap M.J. den Toonder

**Affiliations:** ^1^ Department of Mechanical Engineering Eindhoven University of Technology Eindhoven The Netherlands; ^2^ Institute for Complex Molecular Systems Eindhoven University of Technology Eindhoven The Netherlands; ^3^ Department of Biomedical Engineering Eindhoven University of Technology Eindhoven The Netherlands

**Keywords:** artificial cilia, gelatin methacryloyl hydrogel, live‐imaging, mechanotransduction, MG‐63, siloxanes

## Abstract

Dynamic mechanical stimulation plays an important role in determining the function and health of cells and tissues, and it is therefore highly relevant to study the real‐time response of cells to time‐dependent forces. We introduce a platform for providing controllable dynamic mechanical stimulation to single cells, suitable for investigating large cell populations and enabling live cell imaging, and we present proof‐of‐principle experiments that demonstrate the platform's capabilities. Cells are cultured on a hydrogel surface with magnetic artificial cilia made from a magnetic elastomer using a tailored micromolding process. The cilia are actuated with an electromagnet integrated with an in‐incubator fluorescent microscope. We show that cells attach to the cilia and exhibit widely different morphologies than cells on flat surfaces. Cellular forces involved can be estimated by measuring cilia deflection. We demonstrate that cells can be exposed to continuous dynamic forces by cilia actuation and that their response can be monitored by real‐time observation of Yes‐Associated Protein (YAP). These experiments indicate rare events of mechanotransduction due to cilia actuation, but the low response prohibits drawing final conclusions about the biological response. Our artificial cilia‐based platform offers new opportunities for studying mechanical cell stimulation in real time and understanding dynamic mechanotransduction.

## Graphical abstract

This paper introduces a platform based on magnetic artificial cilia for providing controllable dynamic mechanical stimulation to single cells, suitable for investigating large cell populations and enabling live cell imaging. Proof‐of‐principle experiments show that cell morphology is strongly influenced by the artificial cilia, that cellular forces can be quantified, and that cellular responses can be monitored real time while actuating cilia.

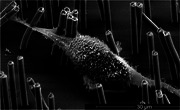

## 1 Introduction

Mechanical stimuli have a profound impact on the health and function of biological tissues, cells, and diseases. For example, compressive stresses on bones regulate bone density [1], stem cell behavior is steered by mechanoresponsive calcium signaling [2] and the survival rates of cancer patients can benefit from physical fitness training [3]. Therefore, the fundamental study of this relationship between mechanical stimuli and cell behavior can yield valuable insights for the development of cell‐based therapies, and for combating diseases.

Since the 1980s, the term mechanotransduction has been used to describe all processes in living cells that are associated with the conversion of mechanical stimuli to biochemical responses [4]. Mechanotransduction processes occur in many different tissues and throughout the cell itself. Furthermore, these processes take place on a range of varying time scales, making mechanotransduction a dynamic phenomenon. Therefore, investigating mechanotransduction in various domains of magnitude and frequency is important, as is the real‐time monitoring of cell behavior to obtain time‐dependent information of cellular processes. Many different experimental techniques have been reported, addressing mechanotransduction processes at various force magnitudes, frequencies, and time scales [5]. From literature, we identify three main principles used for dynamic mechanical stimulation of cells, each with their own strengths and limitations. The first and most common are cell stretching systems using elastomeric [6, 7, 8] or hydrogel [9, 10] substrates to stretch the cells on top or embedded in the material. These systems are typically accessible, cheap, and can be easily tuned to vary actuation magnitude and ‐ to an extent ‐ frequency. Real‐time imaging is often challenging with stretching systems, as the associated movements are large and can cause the sample to shift out of focus. Second, stimuli responsive materials (SRMs) can be used as an alternative cell culture substrate instead of the elastomeric or hydrogel‐based materials. These materials can change properties in response to light [11], temperature [12], or magnetic fields [13]. The impacted material property can vary from stiffness to topography, introducing an interesting versatility. The spatial transformations in SRMs are typically small enough to allow real‐time monitoring, at the cost of a typically low maximum actuation frequency and limited tunability in actuation magnitude. Third, methods based on atomic force microscopy (AFM) offer a high degree of control over actuation magnitude and frequency. While AFM has mostly been used to measure mechanical properties of cells [14], its use for active mechanical stimulation or manipulation of cells has been demonstrated [15, 16]. Some reports have also shown AFM‐based cell stimulation monitored in real time [17, 18], but due to environmental limitations for the effective operation of AFM systems, long duration experiments remain a challenge. In summary, for established dynamic cell stimulation techniques, the combination of controllable actuation with real‐time imaging for longer durations remains a challenge.

Here, we introduce a mechanical cell stimulation platform based on magnetic artificial cilia (MAC), magnetically actuated microscopic pillars, to fill this gap, and we show its capabilities using proof‐of‐principle experiments. Inspired by biology, where cilia carry out diverse and unique functions, we aim to achieve some of this functional diversity in MAC‐based microsystems. Micropillar substrates have been used to study biomechanical responses by magnetic actuation of Poly‐dimethylsiloxane (PDMS) micropillars using a locally placed magnetic tweezer [19]. These systems are capable of applying local stimuli and allow real‐time imaging, but they still face challenges regarding precision of actuation and the volume of cells that can be stimulated simultaneously. Recently, a platform has been introduced for studying mechanotransduction of single cells cased on magnetic hydrogel micropillars made with two‐photon cross‐linking [20]. This platform enables controllable dynamic actuation using a rotating permanent magnet, but it does not allow for real‐time imaging. Here, we show a MAC platform that can be deployed with precisely controlled stimulation magnitude and frequency on hundreds of cells simultaneously, without compromising on either experimental duration or real‐time imaging capabilities. The advantages over previous approaches are due to the unique combination of multiple aspects, namely the ability to apply dynamic forces on single cells on a subcellular scale, the possibility of real‐time cell imaging, and the capability to characterize large numbers of cells in one experiment. We demonstrate these features in this paper with proof‐of‐principle experiments. In‐depth biological analyzes, which are out of scope of the present study, will be the topic of future investigations. First, we discuss the design choices made for the artificial cilia and the magnetic actuator. Next, we show the platform's bio‐compatibility through an assessment of the viability and degree of attachment of MG‐63 cells as a result of MAC actuation. Furthermore, we explore MG‐63 morphology as a result of MAC mechanical stimulation onto the cell membranes. Finally, we show the MAC platform's unique capability to be used for real‐time monitoring of live cells during the magnetic actuation, using Green Fluorescent Protein‐tagged Yes‐Associated Protein (GFP‐YAP) transduced human dermal fibroblasts (HDFs). All of these features combined establish this platform as a useful new tool for the study of dynamic mechanotransduction.

## 2 Results and Discussion

### 2.1 Design and Validation of the Actuator and the MAC Device

**FIGURE 1:**
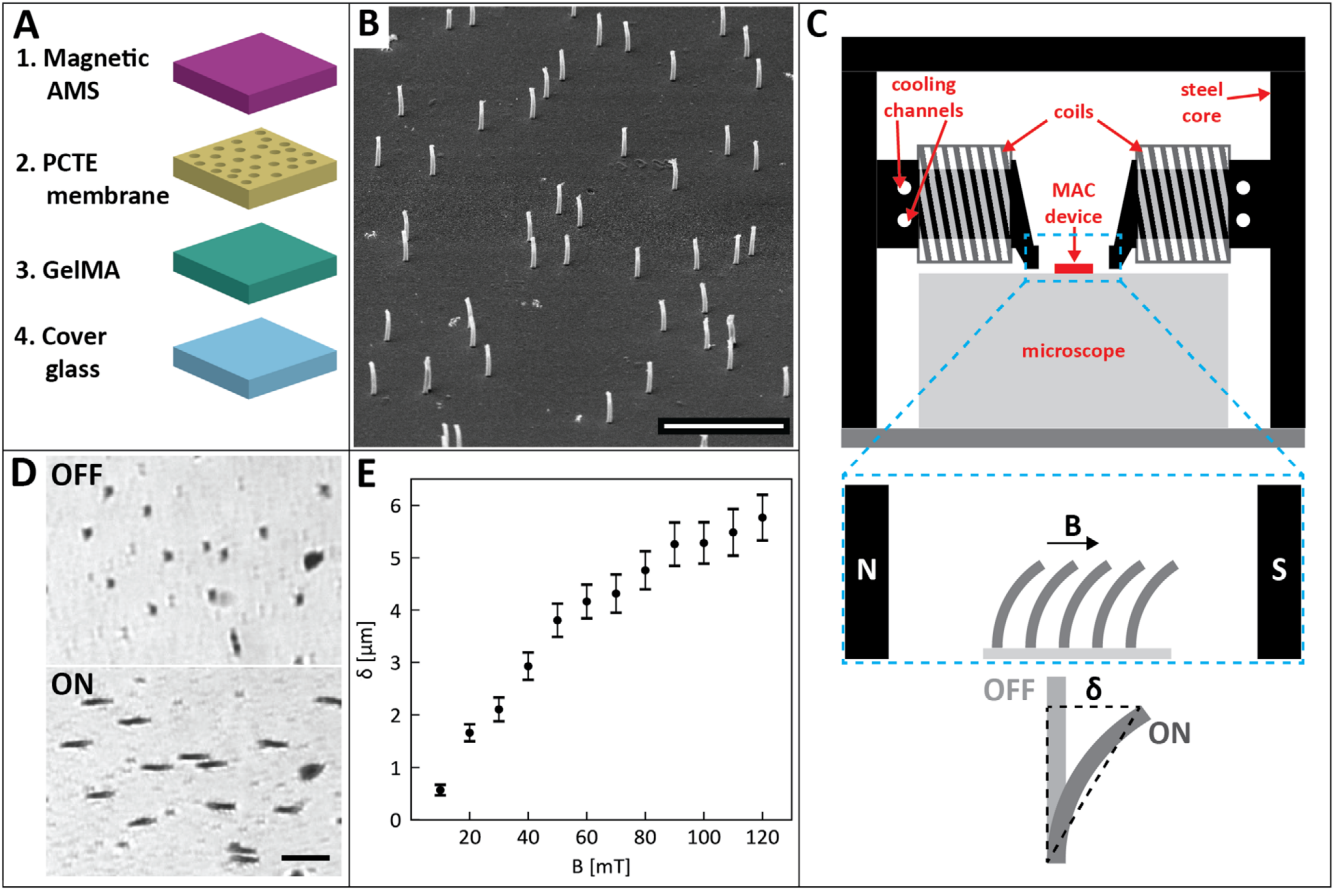
(A) Layer‐by‐layer make‐up of the MAC device. The thickness, pore diameter and areal pore density of the PCTE membranes can be varied to control MAC length, diameter, and areal density, respectively. (B) Scanning electron micrograph of artificial cilia, with length 23μm, diameter 2μm and areal density 100.000${\mathrm{cm}}^{-2}$. Scale bar represents 50μm. (C) Schematic front view of the electromagnetic actuation setup. The left and right coils can be controlled separately to produce a maximum combined field strength of 125mT. The steel core is designed to yield a homogeneous magnetic field at the site of the MAC device. The schematic below indicates the cilia bending under application of a magnetic field with tip deflection δ. Details of the setup are shown in the figures in the Supporting Information. (D) Bottom view of the MAC switched off (top) and switched on (bottom). Scale bar represents 20μm. (E) Measured MAC tip deflection as a function of the magnetic field magnitude, *B*. N = 267 artificial cilia from the same MAC device per data point. Error bars show the 95% confidence intervals.

The MAC devices are made from poly‐(aminopropyl‐methyl) siloxane (AMS), a hydrophobic siloxane polymer, into which magnetite particles are embedded (Figure 1A), using a unique micro‐molding technique described in Section 4.4. We used commercially available polycarbonate track‐etched (PCTE) membranes as a template for micro‐molding the artificial cilia. These membranes can have varying thickness, pore diameter, and pore density, which results in corresponding MAC length, diameter, and areal density. The reason for the use of PCTE membranes is the fact that we fabricate artificial cilia of (sub‐)micron diameters with high aspect ratios (∼11), which prohibits the use of more traditional micro‐fabrication methods involving either (de)molding or additive manufacturing. PCTE membranes combine the desired dimensions for the MAC device with a solubility in chloroform, which we can exploit for demolding the finished MAC device (Figure S1D). A consequence of the ion track‐etching method used to create these templates, however, is that the placement of the pores cannot be exactly controlled, resulting in random placement of the artificial cilia with the areal density giving a measure of the average spacing between the structures (Figure 1B). After the magnetic siloxane is cured, the PCTE membrane containing it is placed on top of a layer of methacrylated gelatin (GelMA) and the artificial cilia and GelMA are fused together by UV‐crosslinking the GelMA (Figure S1). The hydrogel GelMA is already established as a suitable cell culture material, and it allows good MAC actuation in aqueous environments (Figure 1D,E), which makes it an ideal material for our devices.

We next focus on the magnetic actuation setup, which has to finely control MAC deflection. From previous work, we know that the MAC achieve (near‐)maximum bending in a magnetic field with a flux density of around 130mT [21]. To achieve this magnetic control, we can either 1) move a permanent magnet closer or further from the MAC, or 2) use an electromagnet which generates magnetic flux density that increases linearly with electric current. Here, we have opted to use an electromagnet for MAC actuation (Figure 1C). This decision is motivated by the fact that electromagnets offer greater precision in controlling magnetic field strength (Figure 1E), as well as better homogeneity of field strength and direction over the actuated area (Figure 1C; Figure S3). In comparison, permanent magnets exhibit heterogeneity in both the magnetic field strength and direction depending on the position relative to the magnet, significantly complicating MAC actuation using permanent magnets. Furthermore, keeping a clear line of sight to enable microscopy during actuation is more easily achieved using an electromagnet, since we can position the electromagnet coils to the sides of the actuated volume, keeping the z‐axis clear.

Combining the climate control required for long duration experiments with real‐time imaging capability poses a further design restriction. One way of achieving this combination is by integrating an electromagnetic setup in a climate‐controlled microscope. The main advantages of this would be the high resolution and more flexible fluorescent excitation and emission settings. However, these microscopes typically have only small closed‐off climate‐controlled volumes, with typical side lengths of tens of centimeters and a few centimeters in height, meaning that the space available for a custom setup is limited. This would force us to miniaturize the electromagnetic actuator, adding extra design challenges, for instance in the prevention of overheating the electromagnetic coil. Instead, we opted to incorporate an AxionBio Cytosmart LUX3FL microscope into our design, allowing us to run the complete assembly inside a standard‐issue incubator (Figure 1C). While the possible imaging resolution and the number of fluorescence channels in this design are relatively low, the *LUX3FL* still allows the use of a quite diverse set of live‐cell dyes with its imaging channels.

The magnet is controlled using LabView software, and it is wired to enable individual control over both coils. This software is what limits the actuation frequency, sending updates to the magnet at 60Hz. This frequency limit is relatively high compared to most in vivo mechanical stimulation frequencies, meaning that it meets most experimental needs. The limits of actuation magnitude are due to the copper wire used in the coils, which allow us to run currents up to 20A. This will enable the generation of magnetic fields up to ∼100mT according to our simulations (see Figure S3B for a detailed description). Experimentally, we measured magnetic flux densities up to 125mT (Figure 1E). The simulations also show us the spatial homogeneity of the generated magnetic field, with at most 10mT difference in flux density in the central 10mm region between the poles of the steel core (Figure S3B).

The last issue requiring attention when using an electromagnet is the coil heating up due to electric resistance when a current is run through it. To prevent either the magnet from overheating or the temperature in the culture area rising above 37 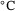, we need to cool the magnet during the experiments. To do this, we added channels that were drilled through the back of the steel cores (Figure 1C) to be used as a water cooling channel; the layout of the cooling channel is shown in Figure S4A. We ran a COMSOL Multiphysics simulation to verify whether the cooling channel dimensions and placement would provide a feasible way of keeping the maximum temperature at 37 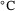 Figure S4B. This simulation assumes that the magnet is running at full power, meaning that the core receives 44W of heating. The cooling water temperature is at room temperature (295.15K) flowing at 12 m s^−1^. The pumping and temperature management are done using a commercially available recirculating cooler, which is capable of maintaining the required water temperatures using a flow rate of 8 m s^−1^ while maintaining a lower water temperature than the simulated 295.15 K. Nevertheless, on top of this, the aforementioned control software is equipped with a cutoff temperature of 50 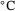 to prevent any damage to the coils in case of a cooling failure. We carried out experiments to support the numerical simulation. The setup was operated at maximum power in the incubator, but without cell culture, and the temperature within the incubator was measured. The measured temperature was not affected by the setup operation, and it remained stable. We are therefore confident that any observed cellular behavior is not due to temperature effects.

### 2.2 Biocompatibility of the MAC Device

**FIGURE 2:**
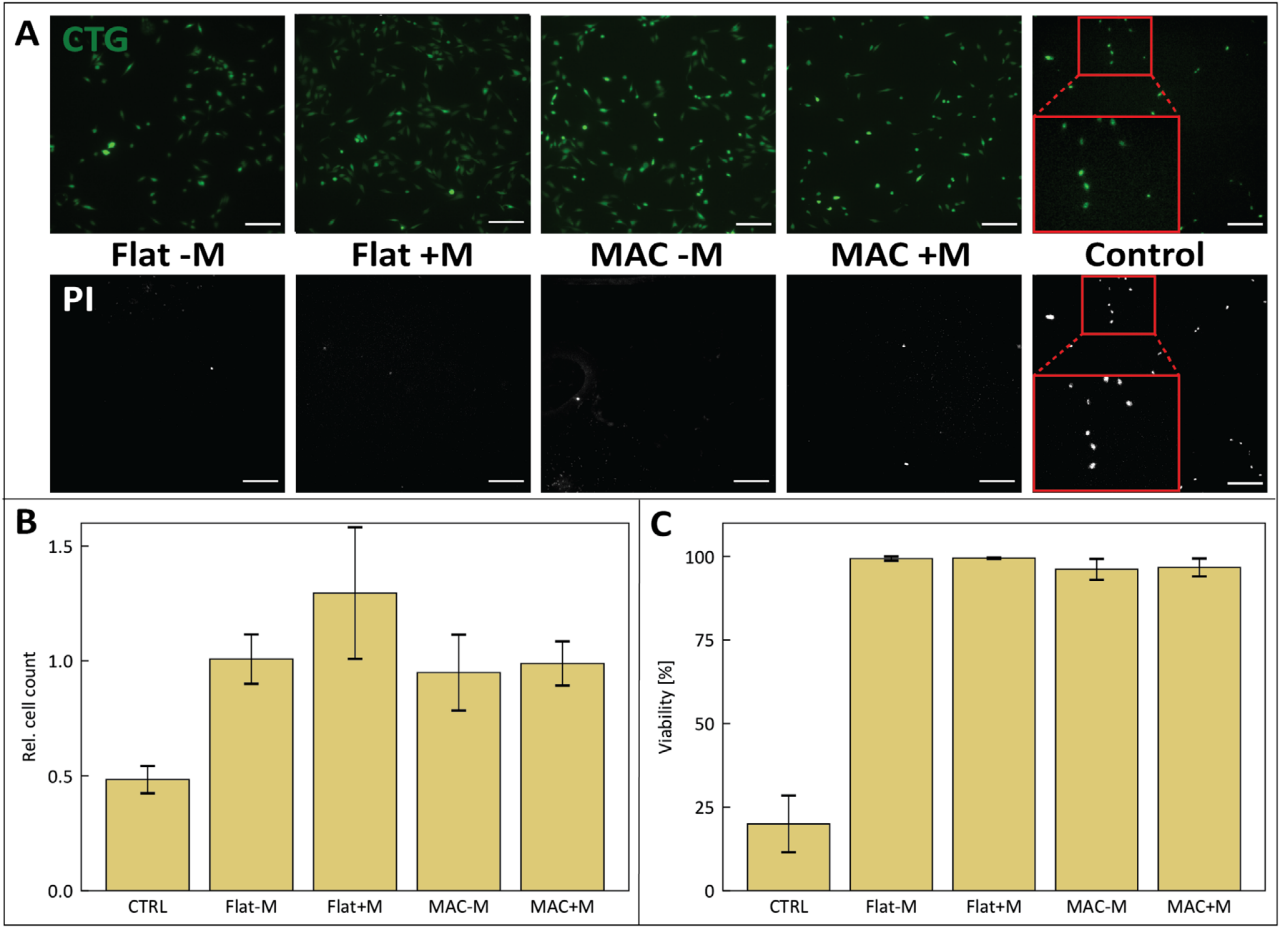
(A) Representative fluorescent images showing the CTG staining (highlighting all attached cells) and PI staining (highlighting dead cells only), for the flat GelMA surfaces without (Flat‐M) and with (Flat+M) magnetic field, the MAC devices without (MAC‐M) and with (MAC+M) magnetic actuation and the control consisting of MG‐63 cells killed with 0.5% Triton X‐100 on glass surfaces. Scale bars represent 150μm. (B) Cell count after treatment, relative to the count before treatment, showing the degree of cell attachment. (C) Cell viability measured as the percentage of live cells in the total count after treatment. (B/C) Showing means of N=3 replicates, attachment and viability percentages were calculated from populations of cells between 5000 and 10.000 cells. Error bars show standard deviations.

We assessed the biocompatibility of the MAC device by monitoring cell adhesion and viability, both as a result of MAC actuation and of the magnetic field itself. For this, we performed a CellTracker‐Green (CTG) staining as a live dye on MG‐63 cells before seeding them on MAC devices. The devices were either actuated (MAC +M) or left stationary (MAC ‐M) for a duration of 2 h. We have included controls, exposing the cell culture on flat, non‐magnetic GelMA substrates to either a magnetic field with the same strength (Flat +M) or no magnetic field (Flat ‐M). Lastly, we included a positive control consisting of cells killed using Triton X‐100 on a glass surface. The actuation for the MAC devices and for the “Flat +M” control was set as a triangle wave with an amplitude of 125mT and a frequency of 0.5Hz. After the 2 h experimental treatments, the cells were stained with propidium iodide (PI) to visualize dead cells. We calculated cell viability as the percentage of live cells, and cell attachment as the ratio of cells per unit area left after experimental treatment compared to the before‐treatment count. In total, we performed three replicates of the experiment, and the results are shown in Figure 2. Our results clearly show that neither the attachment nor the viability of these MG‐63 cells was adversely affected by either the presence of the magnetic field or the MAC actuation. We note that these 2‐h experiments are not conclusive about the long‐duration biocompatibility of the device; notwithstanding are the viabilities under all conditions very close to 100%, which gives good confidence about the biocompatibility for longer durations. It is vital to also note the importance of the inclusion of a control consisting of cell experiencing a magnetic field on a flat culture surface. Existing literature reports that magnetic fields alone can have an effect on cytokine secretion [22, 23, 24, 25] or differentiation [26], meaning that phenotypical readouts resulting from MAC actuation should always be compared to the effects of the exposure to merely the magnetic field. Additionally, these control experiments rule out any influence on MG‐63 cell attachment and viability of the potential temperature change that may be induced by the magnetic field generation.

### 2.3 Topographical Mechanical Cues of Static MAC

**FIGURE 3:**
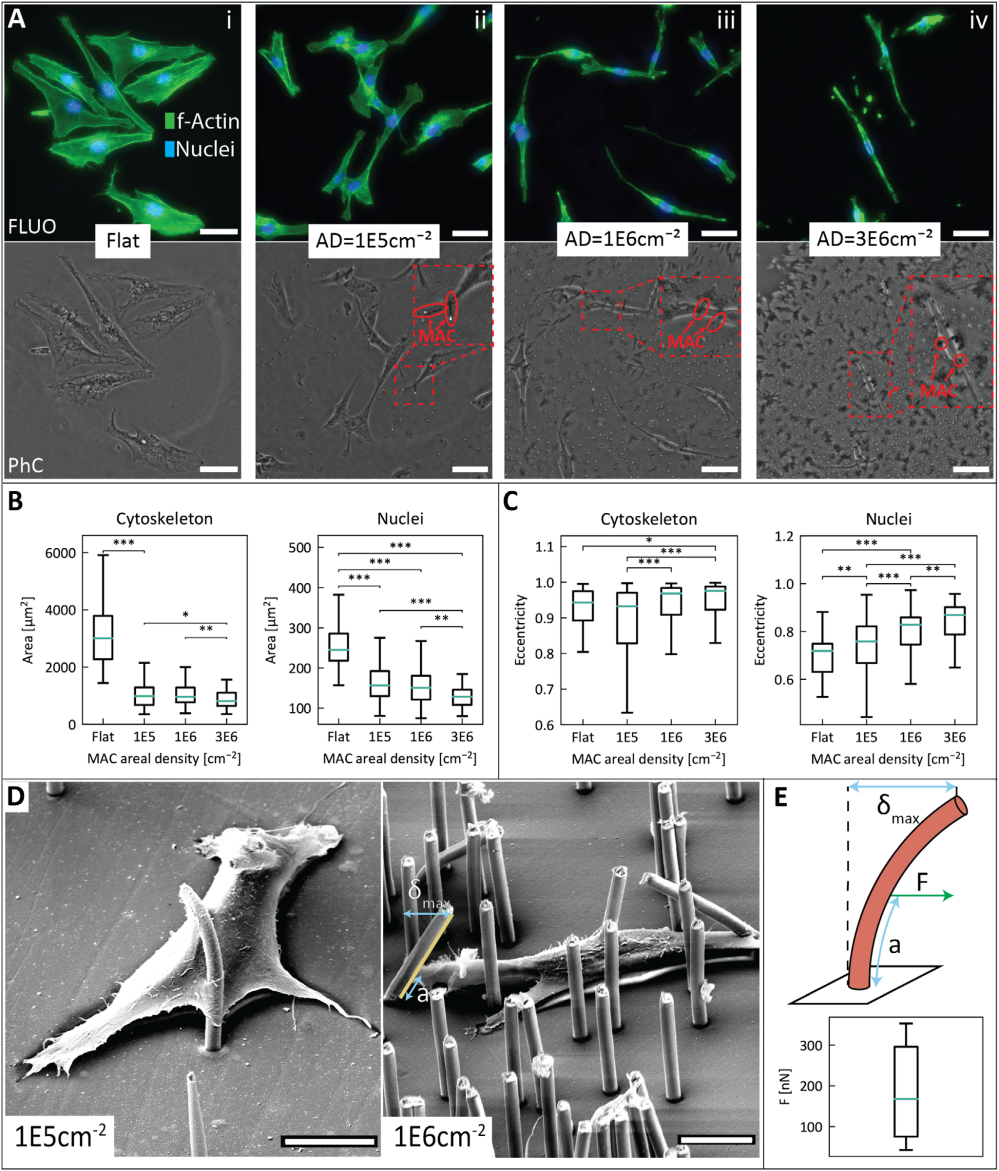
(A) Fluorescent (top) and phase contrast (bottom) micrographs illustrating the MG‐63 cel morphology on varying areal densities (AD) of MAC compared to a flat AMS substrate. Insets in the phase contrast images highlight the MAC. Scale bars are 50μm. (B) Box plots showing quantitative comparison of projected areas of the cytoskeleton and the nuclei of MG‐63 grown on flat AMS substrates and MAC devices with increasing areal densities, medians are shown in green. (C) Box plots showing quantitative comparison of eccentricity of the cytoskeleton and the nuclei of MG‐63 grown on flat AMS substrates and MAC devices with increasing areal densities, medians are shown in green. The significance asterisks in B/C represent pairwise comparison p‐values with *: p<0.05, **: p<0.01, ***: p<0.001. N = 80 ‐ 148 cells per condition in total. (D) Scanning electron micrographs of an MG‐63 cell on a MAC surface with low areal density (left) and medium areal density (right). Scale bars are 10μm. (E) Schematic illustration of the estimation of the MG‐63 pulling forces from the measured MAC tip displacement (top) and the obtained values (bottom, N=6).

Surface topography impacts cell behavior, which can already be inferred from cell and nuclear morphology [27]. Our artificial cilia, when stationary, form a topography of soft pillars. Therefore, to fully characterize the impact made by the MAC on cell behavior, we examine cell and nuclear morphology resulting from culturing cells on static MAC substrates. We cultured MG‐63 cells on different MAC areal densities to vary the collective bending stiffness of the MACs, as well as the space cells have between the artificial cilia. Figure 3A shows fluorescent and phase contrast images of the MG‐63 cells on these surfaces, where the artificial cilia can be seen in the phase contrast images. The artificial cilia appear as brighter spots when upright, while appearing darker when viewed at an angle. This is caused by the additional phase differences of the light passing through a curved surface to the detector as opposed to light passing through a flat surface. From these images, the random distribution of the artificial cilia is evident, which is due to the nature of the PCTE membranes used as templates as described earlier; the consequence is that cell‐cilia interactions cannot be fully regulated. This apparent flaw may also be considered an advantage, since the statistical distribution of the MAC locations may also result in a richer behavior between individual cells from which broader conclusions may be drawn for the whole cell population. We cultured MG‐63 cells for 24 h on substrates of stationary MACs with low ($10^{5}{\mathrm{cm}}^{-2}$), medium ($10^{6}{\mathrm{cm}}^{-2}$) and high ($3*10^{6}{\mathrm{cm}}^{-2}$) areal density. Subsequently, we stained the cells with DAPI and phalloidin to visualize their nuclei and cytoskeletons, respectively. To mount the MAC devices for microscopy, we designed PDMS sample chambers (Figure S5) to prevent the artificial cilia from collapsing under the cover glass. These mounting devices leave 50–100μm between the sample and the objective. From the obtained fluorescence images, we extracted morphological features of individual cells using the open software CellProfiler. Analyzing cellular and nuclear morphology can provide indications of changing phenotypes. We used two of these features to characterize the morphology of the actin cytoskeleton and nucleus: projected area, and eccentricity. Eccentricity is normalized between zero and one, with higher eccentricity values representing a more elongated shape. On a flat AMS surface, MG‐63 cells are spindle‐like with a length of ∼100μm (Figure 3A). Furthermore, we observe small filamentous protrusions of the actin cytoskeleton. These characteristics are typical for MG‐63 cells in vitro [28, 29]. Additionally, we can see that an increasing areal density of artificial cilia causes the projected area of both the cell and nucleus to shrink. Also, both the cytoskeleton and nucleus take a more elongated shape (Figure 3B/C). The cell elongation observed on MAC surfaces can be explained by the phenomenon of contact guidance [30], and is in line with existing literature describing morphological effects of microtopographies [31]. The edges of the artificial cilia form topographical features along which the cells align themselves. With the space between the artificial cilia becoming smaller with increasing areal density, cells inevitably will reduce their projected area and elongate to fit in the space in between the artificial cilia. Finally, for the MAC devices themselves, we observe that higher areal density artificial cilia devices often stick together at the tips, rendering them immotile for actuation purposes.

To investigate whether the MG‐63 cells attach and interact directly with the MAC, we performed scanning electron microscopy (SEM) on MG‐63 cells cultured for 24 hours on MAC surfaces with areal density $10^{5}{\mathrm{cm}}^{-2}$ and $10^{6}{\mathrm{cm}}^{-2}$. Since samples need to be dried before performing SEM, and the artificial cilia will collapse if the surrounding liquid evaporates, we performed critical point drying (CPD) to dry the sample while preventing MAC collapse. We note, that the SEM images are for morphology or structural observation only, because the dried state obviously does not represent the in situ aqueous environment. From the images of MG‐63 cells on the low‐density MAC surface, we can see that the cells form strong attachments to the MAC despite the abundance of space available to avoid them (Figure 3D), even partly enveloping artificial cilia. On MAC surfaces with an areal density of $10^{5}{\mathrm{cm}}^{-2}$, the number of cilia contacted per cell ranges from 0 to 6, while any higher areal MAC density surface does not have any cells without MAC interactions.

We used the open software *ImageJ* to measure the MAC tip displacement δ and the cell attachment height *a* from the SEM images (Figure 3E), taking the 45° sample orientation into account. Interestingly, while most cell‐cilia connections are close to the base substrate, we also observe the MG‐63 cells attaching to cilia much higher up (Figure S7D,F). Using the obtained $\delta_{max}$ and *a* values, we calculated the force exerted by the MG‐63 cells onto the artificial cilia using the following equation for bending cantilevers:

(1) $$F=\frac{6EI\delta_{max}}{a^{2}\left(3L-a\right)}$$

where *E* is the material's Young's modulus, *L* is the length of the cilium, *a* is the distance from the cilia base to the location of force application, and *I* is the structure's cross‐sectional moment of inertia given for circular cross‐sections by:

(2) $$I=\frac{\pi r^{4}}{4}$$

where *r* is the cilium radius.

For our calculations, the Young's Modulus of the MAC is taken from the results of our previous work [32]. It is important to note that the error on the calculated force values is likely large, since the SEM images were taken in a vacuum rather than the medium present during culture, and due to inaccuracies in measuring pixel distances. Furthermore, our calculations assume that the artificial cilia were upright before the initiation of the cell‐cilium contact (Figure 3E), and while this does seem close to reality (Figure 3D(right)), there are likely cases of artificial cilia having an initial tilt without cells present. We also assume that the GelMA layer underneath the artificial cilia does not locally deform; such deformation is not observed in the SEM image in Figure 3D, nor in the images shown in the Figure S7, but it cannot be ruled out completely. Nevertheless, these rough calculations suggest that the magnitude of the force MG‐63 cells exert on the artificial cilia falls within the range of 10
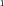
–10
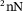
(Figure 3E), which is in line with reports from literature [33]. The fact that the MG‐63 cells adhere to the MAC with a sufficient mechanical coupling to generate these forces proves the practical feasibility of magnetic actuation of the artificial cilia for mechanotransduction experiments, thereby meeting an essential requirement for further experimental use. Obtaining further control over the exact anchoring height of the cells on the artificial cilia could be desirable, since this would make the system's force output onto the cells more predictable. This might be possible by adding additional geometrical features onto the MAC. However, with the current fabrication process, this is not possible, because the MAC shape is dictated by the cylindrical geometry of the track‐etched pores in the polycarbonate membranes. Alternatively, one could selectively coat parts of the artificial cilia to create preferential adhesion sites, although gaining sufficient spatial control over the application of such coatings would be challenging at the length scale of this system.

### 2.4 Dynamic Mechanical Cell Stimulation using the MAC Device

**FIGURE 4:**
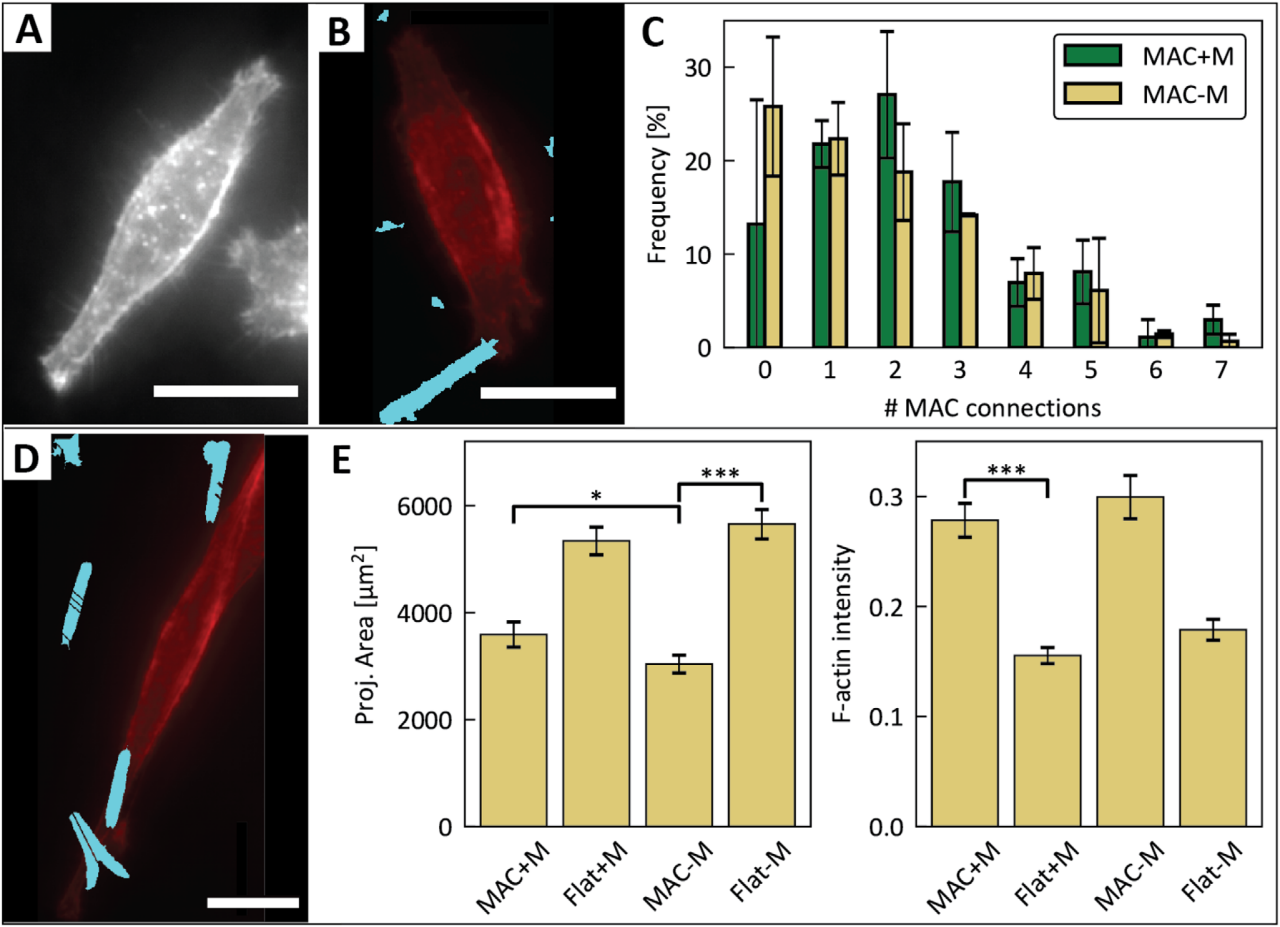
(A) Representative f‐actin staining of a MG‐63 cell on a flat GelMA surface without applying a magnetic field. Scale bar is 20μm. (B) Representative f‐actin staining of a MG‐63 cell on a MAC device without magnetic actuation. The cyan overlay indicates the location of the artificial cilium (1 cell‐MAC connection). Scale bar is 20μm. (C) Distribution of the number of MAC‐connections formed per cell, comparing actuated MAC (MAC+M) samples with static MAC (MAC‐M) samples. Means are calculated from the cell counts of three biological replicates, with error bars showing standard deviations. (D) Representative f‐actin staining of a MG‐63 cell on a MAC device which has been actuated for 2h. The cyan overlay indicates the location of the artificial cilia (four cell‐MAC connections). Scale bar is 20μm. (E) Left: projected cell area for three biological replicates of cells cultured on flat GelMA surfaces and MAC devices, both with a non‐magnetic control (Flat‐M, MAC‐M) and actuated with an oscillating magnetic field (Flat+M, MAC+M). Right: Median intensity per cell of the f‐actin staining, comparing the same experimental circumstances. Error bars show 95% confidence intervals. Statistical significance asterisks show p‐values with *: *p* <0.05, **: *p* <0.01, ***: *p* <0.001.

As mentioned in the previous section, cell morphology is correlated with cell function. Thus, to show the biological effect of MAC stimulation, we investigated MG‐63 morphology after 2 h of actuation at 120mT, 0.5Hz. Again, we added controls to isolate possible effects of either the magnetic field and possibly associated temperature effects, or the stationary MAC on our observations. The results are shown in Figure 4. For the projected cell area after actuation, we can discern a significantly lower area for cells on MAC surfaces than cells grown on flat surfaces (Figure 4E). However, comparing the actuated MAC devices with the stationary control, we see a small increase in area for actuated MAC devices. While it is statistically significant, it is unclear whether this difference has a significant biological impact. We have similarly analyzed the intensity of the f‐actin staining itself, since this can give information about the degree of reinforcement of the cytoskeleton. While it is interesting that the f‐actin intensity increases significantly for MG‐63 cells cultured on MAC devices compared to flat surfaces for both actuated and passive circumstances, we do not see a difference resulting from the actuation of the artificial cilia (Figure 4E).

Looking at the cells during actuation (Figure S8B), we see that the artificial cilia stop moving when cells are connected to them. Furthermore, we can see that the cells do not all form the same number of connections with artificial cilia (Figure 4C), following from the fact that we currently have little control over the way in which the cells form connections to the MAC. We have counted and analyzed the frequency at which cells tend to form specific numbers of MAC‐connections ($n_{c}$). Unsurprisingly, higher number of cilia connections occur less frequently. However, there are no statistically significant differences (Table S1) of the occurrence of any specific $n_{c}$ between actuated or stationary devices. While this variability in $n_{c}$ seems like a liability at first glance, we can turn it into an asset by separating the cell populations by the $n_{c}$ per cell. Effectively, this adds an internal screening potential to the MAC device, offering different actuation conditions within one device. The trade‐off is that the sample size per experiment decreases, as we would be comparing sub‐populations on a device. However, with each device providing space for around 10.000 cells, these sub‐populations would still be in the order of hundreds of cells.

Overall, our results suggest that we have not applied sufficient stimuli to trigger a measurable mechanoresponse in MG‐63 cells. The two contributing factors to this effect are the MAC actuation force and the duration of the stimulation. While literature states that mechanoresponsive morphology changes can be observed after dozens of minutes [34], other reports use time scales up to (but not necessarily limited to) 72 h [35]. Therefore, we think it is worthwhile to increase the duration of MAC actuation for the effective induction of mechanoresponses in future studies. Furthermore, we acknowledge that the exerted force onto the cells during magnetic actuation is subject to an unknown variability, stemming from the observation of a spread in MAC tip deflection upon application of a magnetic field (Figure 1E). Lastly, the exact stimulation force on a cell depends on the anchoring height of the cell onto the artificial cilium. Therefore, attempts to control the location of cell adhesion onto the MAC as described in the previous section should significantly increase the consistency and reliability of the produced results.

**FIGURE 5:**
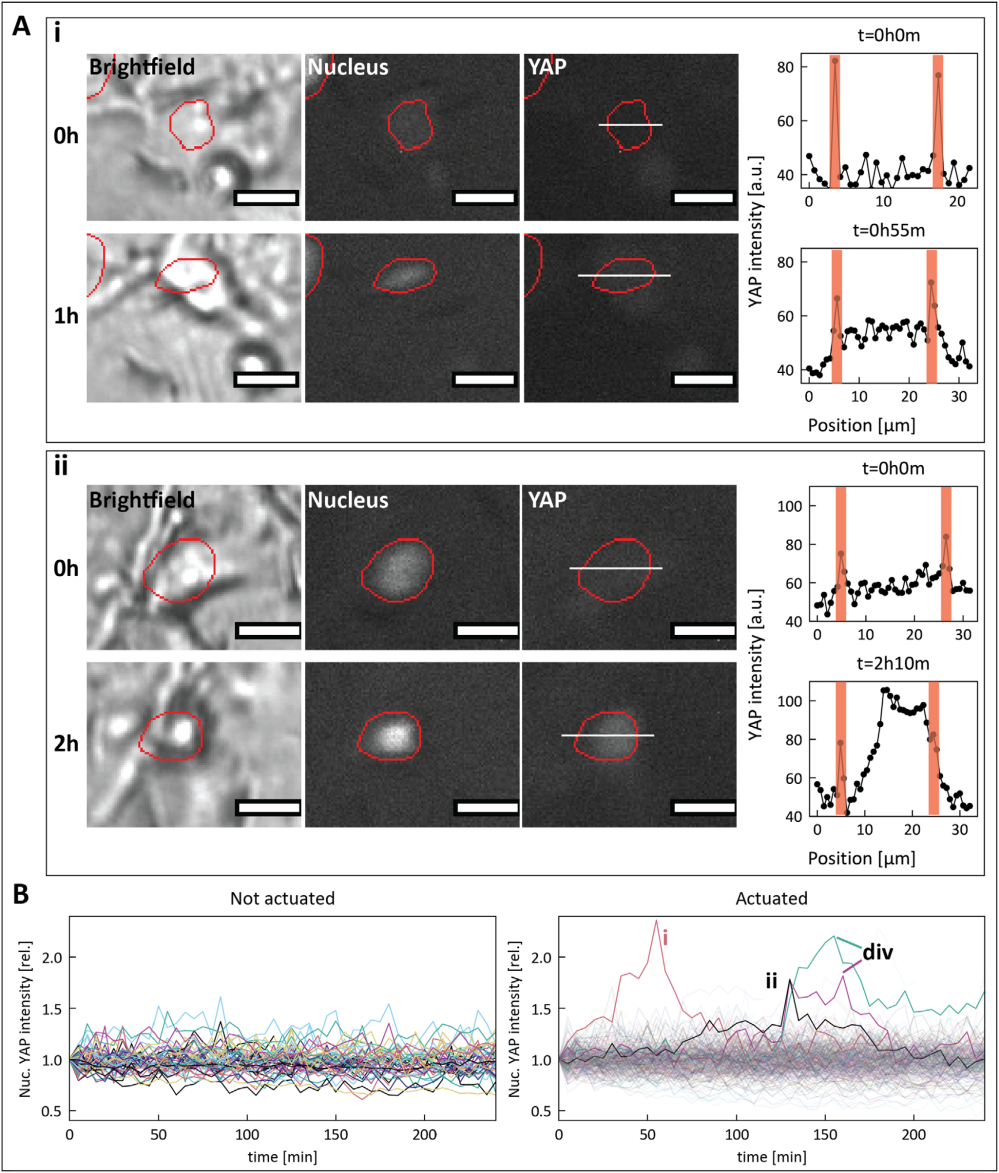
(A) Brightfield (left), nuclear staining (middle) and YAP staining (right) images of two HDF epithelial cells ((i) and (ii)) from the actuated MAC device, with red lines indicating the segmentation performed by the CellProfiler pipeline. Line plots with the YAP pixel intensities are given, with red regions marking the borders of the segmented nucleus. Scale bars are 20μm. **(**B) Mean YAP intensity per nucleus over time, relative to the starting mean intensity of the same nucleus, for both non‐actuated (left, 52 cells) and actuated (right, 319 cells) MAC devices. Cells with more than 1.7‐fold YAP increase in the actuated MAC device are marked ('i', 'ii', corresponding to the cells shown in panels (A) and (B) and 'div'), where the 'div' marking denotes cells undergoing a division.

To demonstrate our platform's compatibility with real‐time imaging, we have transduced HDF epithelial cells with a GFP‐YAP label, and live‐stained their nuclei (SPY555‐DNA). We cultured the GFP‐YAP SPY555 HDFs on a MAC device, actuating them for 4 h at 120mT, 0.5Hz. The expectation for this experiment is that the dynamic mechanical stimulation provided by the MAC induces transportation of YAP to the nuclei, where it is known to act as a transcriptional regulator [36, 37]. Every 5 min, we took images on the brightfield channel, as well as both fluorescent channels (Figure 5A). We used a custom‐built CellProfiler pipeline to segment the nuclei regions from their respective fluorescent images using, as well as to measure the YAP image intensity inside the same regions. We note that there is a discrepancy between the number of segmented objects between the stationary MAC control sample and the actuated MAC sample (Figure 5B). For the actuated MAC, more nuclei were stained more successfully than on the control sample (Figure S9), somewhat lowering the statistical power of these results. On the successfully segmented objects, we used the open source *TrackPy* python module to link the nuclei objects to their corresponding nuclei in each subsequent image over time. This allows us to study the time‐dependent change in fluorescent signal per individual cell. In Figure 5B, we show the mean YAP signal for each segmented nucleus over time, on stationary and actuated MAC. Each nucleus' YAP signal has been normalized to its intensity at t = 0, to allow a straightforward fold‐change analysis of the results.

On the actuated MAC, there are four remarkable objects with a rise in YAP intensity greater than any object on the stationary MAC. Upon examination of the time‐lapse images, we found that two of these cells were dividing at the time of the measured YAP increase. Due to changing surface area/volume ratios of mitotic cells, we excluded these cells from analysis. The remaining two cells are shown in detail in Figure 5 Ai, ii, and here we clearly see an increase in nuclear YAP concentration which is not observed for any non‐actuated cells. This suggests the presence of sufficient mechanical stimulation by MAC actuation for these two cells to invoke a response. However, with two out of a total of 319 cells showing detectable YAP increases within the 4 h actuation time span, we have insufficient statistical power to draw significant conclusions and more investigation will be needed to assess and improve the efficiency of cell stimulation, staining and imaging. Moreover, the low signal intensity on the YAP images decreases the sensitivity of the assay in general.

To summarize, we are able to maintain a real‐time view of the cells while mechanically stimulating them using the MAC devices. Also, we can track and correlate fluorescent signals over time. Our first tests using a GFP‐YAP transduced HDFs cell line therefore provide a first proof of concept for dynamic mechanical cell stimulation. Further optimization of staining or transduction protocols should be pursued to improve the signal. Alternatively, a redesign of the magnetic actuator, aiming to minimize its dimensions, could enable the use of more powerful microscopes to improve image quality and increase the number of possible simultaneous fluorescent signals.

## 3 Conclusions and Outlook

We have developed a platform for providing controllable dynamic mechanical stimulation to living cells based on magnetic artificial cilia (MAC). Taken together, the results of the proof‐of‐principle experiments we present here show that our platform uniquely combines multiple aspects, namely (1) our magnetic artificial cilia (MAC) enable to apply dynamic forces on cells on a subcellular scale; (2) our platform includes an in‐incubator fluorescent microscope, which enables real‐time live cell imaging while mechanically stimulating the cells; (3) the setup enables to characterize a large number of cells in one experiment, providing rich statistical information. Previously published approaches using micropillar substrates for regulating and characterizing cell‐substrate interactions offered one or two of these possibilities but not combined. Our results show that MG‐63 cells cultured on passive MAC attach to the cilia and exhibit widely different morphologies than cells on flat surfaces, and that MAC areal density has a significant influence on cell morphology. Also, by measuring MAC deflection, cellular forces involved can be estimated. We have shown the biocompatibility of the MAC system. Furthermore, we have explored the effects of MAC stimulation on the morphology of MG‐63 cells and found no significant changes in cell area or f‐actin concentrations. It is possible that the forces exerted by the MAC are too small to cause detectable phenotypical changes. Therefore, to try to increase this force, future work could involve increasing the size of the artificial cilia, while keeping in mind the trade‐off between magnetic torque and bending stiffness. Alternatively, new generations of electromagnets can be designed to output stronger magnetic fields. Also, the artificial cilia‐cell interactions have inherent variability due to the random placement of the MAC and the cilia‐cell attachment height; these apparent flaws could be considered as advantages since they provide the opportunity to obain richer statistical information, when properly analyzed. However, to enhance predictability, these aspects can be addressed by using alternative fabrication methods (e.g., photopatterning or magnetic‐field‐assisted alignment before curing) and designs or surface treatments of the MAC, which all are challenging at the length scale of this system, though.

Finally, we have shown the real‐time imaging compatibility of our system using GFP‐YAP transduced epithelial cells during dynamic loading. Even though we observe rare events of mechanotransduction, overall, these results are inconclusive due to a lack of statistical significance. Further optimization of staining or transduction protocols could enhance this significance by offering image quality improvement. We also think that it is prudent to aim to miniaturize the magnetic actuating system to fit inside more powerful microscopes to enable more detailed imaging.

While our experiments show proof‐of‐principle of the platform highlighting its capabilities, future experiments should focus on more in‐depth biological analyses, which were not within the scope of the current investigation. Toward potentially enhancing and understanding the cellular response of the HDF cells, the duration of the experiments should be extended beyond 4 h [35], the magnetic field strength could be increased, GFP‐YAP transduction could be enhanced, and functional connections between cells and artificial cilia should be quantified. The latter can include quantifying the expression and localization of integrins (e.g., αvβ3) and adhesion complex proteins (e.g., vinculin), and correlating the results with MAC areal density and actuation conditions. Beyond the presented studies, our artificial cilia‐based platform offers new opportunities for studying mechanical cell stimulation in real time and understanding dynamic mechanotransduction.

## 4 Materials and Methods

### 4.1 COMSOL Multiphysics Modeling

Our models are fully coupled 3D stationary models.

#### 4.1.1 Magnetic Flux Density

To simulate the magnetic flux density, we defined the geometry given in Figure S10. We performed a stationary study using the Magnetic Fields physics module.

#### 4.1.2 Water Cooling

To simulate the water cooling, we used a simplified model geometry containing only one steel core including the water channel with inflow and outflow boundary conditions. We used the physics modules Heat Transfer in Solids and Fluids, Laminar Flow and Multiphysics; Nonisothermal Flow.

### 4.2 Synthesis of Magnetic AMS

AMS‐162 siloxane copolymer was purchased from *Gelest*. All other reactants were purchased from Sigma–Aldrich (Merck). As a reaction vessel, a modified 100mL glass bottle from VWR was used. The bottle cap was modified with an electric motor driving a glass stirring bar to mix the solution without the need for a magnetic stirring plate, as well as two reagent addition inlets and an inlet and outlet for nitrogen gas flushing. In two clean glass beakers, 1.24g of $FeCl_{2}\cdot 4H_{2}O$ and 0.74g of $FeCl_{3}$ were dissolved separately in 20mL of milliQ pure water. The reaction vessel was cleaned thoroughly with acetone, dried, and rinsed with nitrogen gas before adding both reagents to it and closing the vessel, giving a total volume of 40mL. Next, the solution and reaction vessel volume were purged with nitrogen gas for 20 min. Stirring was brought up to create a vortex while keeping the fluid level stable without splashing. Then, a buret was used to slowly add 20mL of ammonium hydroxide ($NH_{4}OH$) to raise the pH of the solution. As the pH rose, a black precipitate formed, which dissolved as the pH rose further. After stirring the alkaline solution for another 10 min, 2mL of AMS‐162 was slowly added using a syringe. Lastly, all connecting valves were closed, nitrogen influx stopped and the solution was stirred strongly overnight (∼16 h).

After the reaction and coating finished, the stirring was stopped and the vessel was placed on a neodymium permanent magnet to let the magnetite particles sediment. Once the solution had turned mostly clear, the supernatant was gently poured off, ensuring that the black sludge remained in the reaction vessel. The product was washed four times with methanol, adding ∼80mL for the first step and ∼50mL for the remaining steps. For each washing step, during the methanol addition, the glass stirring bar and the sides of the reaction vessel were rinsed to maximize yield. Each washing step included 3 min washing on a shaking plate at 240−300rpm, followed by sedimentation using the permanent magnet underneath the reaction vessel. Next, the product was washed three times in a similar fashion with milliQ pure water. During these washing steps, the solution should yield a black flaky precipitate. Finally, another three washing steps using methanol were performed. After the last washing step and sedimentation / decanting, the product was dissolved in 15mL of chloroform by sonication for 30 min. All of the glassware involved in the reaction and washing of the product was dried out, weighed, cleaned, and weighed again to determine the total loss amount. The dissolved product was transferred to a pre‐weighed glass vial and left on top of a permanent magnet for 30 min to let any uncoated magnetite particles sediment. Subsequently, the magnet was removed and the supernatant transferred to a fresh pre‐weighed glass vial. The transferred solution was sampled (≤1mL) and weighed in a third pre‐weighed glass vial for determining the percentage (wt) of coated particles in the solution. The sample and sediment vials were left to let the solvent evaporate for ∼5 h before determining their dry weights. All of the weights for the sediment, sample, solution, and losses were used to calculate the final yield (Figure S11) and concentration of the product before adding AMS‐162 and the photo‐initiator dicumyl peroxide to tune the solution to the desired concentration. The final solution was sonicated again for 30 min to completely dissolve and mix the final ingredients before leaving the vial open for ∼24 h to allow the solvent to evaporate.

### 4.3 Quantification of MAC Tip Displacement

A custom Python program (Figure S12A) was used to load images of MAC with and without magnetic field present side by side. The distances between the cilia tips in ‘off’ and ‘on’ states were drawn manually using the image as a guide, and the data were analyzed in R before visualization using Python. One MAC sample was used for this analysis, with N = 267 artificial cilia per data point.

### 4.4 Magnetic Artificial Cilia Fabrication

The schematics in Figure S1 show details of the MAC fabrication steps. Polycarbonate track‐etched (PCTE) membranes were purchased from *it4ip S.A*.. Before use, tweezers, glass rods, and glass slides were wiped with a cleanroom cloth containing acetone to remove any dust and impurities. A thin first layer of magnetic AMS (ferrofluid) was applied to the top of a fresh PCTE membrane before curing at vacuum and 120 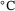 for 1 h and 45 min.

Using a clean glass rod, a thin layer of already prepared GelMA was applied to the top of a clean cover glass, see Figure 1 and Figure S1. The thickness and the elastic modulus of the 5% w/v GelMA layer was ≈5μm and ≈10kPa; the latter is based on literature [38]. Next, the PCTE membrane containing the cured magnetic fluid was placed onto the GelMA layer. The GelMA was cured by exposure to 365nm UV light (100 mJ ${\mathrm{cm}}^{-2}$). The sample was washed twice in chloroform for 2 min each cycle to dissolve the PCTE membrane, before it swells and splits off the top magnetic layer [21, 32], leaving the freestanding MAC behind. The releazed cilia were rinsed in ethanol for 5 min afterward. Finished MAC devices were stored in wells containing de‐ionized water until further use.

### 4.5 MG‐63 Cell Culture

Initial MG‐63 cell line was obtained from *ATCC* (catalog no. CRL‐1427). The cells were cultured in growth medium consisting of α‐Minimum Essential Medium (*GibCo*) supplemented with 10% Fetal Bovine Serum, 100 U mL^−1^ penicillin and 100 μg mL^−1^ streptomycin. Under aseptic conditions, the frozen cells were thawed and immediately transferred to fresh, warm growth medium, at a ratio of 10mL of growth medium to 1mL of cell suspension. To remove the cryopreservative medium, the suspension was centrifuged at 130g for 7 min. The supernatant was replaced with fresh growth medium and the cells were resuspended. Cells were cultured at 37 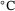, 5% ${\mathrm{CO}}_{2}$ and humid environment. The cells were passaged upon reaching confluence, and MG‐63 cells of passages 10–20 after purchase were used for experiments.

### 4.6 PDMS Coating with Collagen‐I

Collagen type‐I was purchased from *Thermo Fisher GmbH*. 0.5cm x 0.5cm sheets of PDMS were placed in a 24‐wells plate, sterilized by immersion in 70% ethanol for 20 min and washed three times with PBS. Subsequently, the PDMS sheets were immersed in a collagen‐I solution in PBS, diluted to contain 5 μg ${\mathrm{cm}}^{-2}$ of collagen‐I. After 3 h of incubation at room temperature, the PDMS sheets were washed with PBS before use.

### 4.7 Seeding MG‐63 Cells on AMS and AMS‐based MAC Substrates

MAC substrates were cut into pieces of 9mm x 9mm and transferred to a 24‐wells culture plate (*Greiner Bio*) using tweezers. Before placing, the sample was tapped to a dry glass slide to remove ethanol from the underside of the sample, allowing the sample to stick to the well. Directly after sample placement, the well was filled with 0.5mL of 70% ethanol to sterilise the sample. After 20 min, the MAC sample was washed three times with sterile phosphate buffered saline solution (PBS) and a final volume of 0.5mL of growth medium. The submerged sample was incubated for at least 4 h at 37 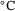, 5% ${\mathrm{CO}}_{2}$ and humid environment to remove possible air bubbles from the substrate. The MAC surfaces were not functionalized with adhesion molecules (like fibronectin or collagen) prior to cell culture; we decided for this approach after analyzing morphology of cells (indicating adhesion) on flat PDMS surfaces with and without a collagen coating, and flat uncoated surfaces of MAC material AMS, which showed similar morphology between cells on collagen coated surfaces and flat AMS surfaces indicating good adhesion. These results are shown in Figure S6.

### 4.8 DAPI and Phalloidin Staining

MG‐63 cells cultured on MAC substrates were fixed using 3.7% formaldehyde solution in phosphate buffered saline solution (PBS) for 20 min. Then, the fixated cells were treated with 1% Triton X‐100 for 15 min for membrane permeabilization. The cells were stained with 1.4 μg mL^−1^ 4',6‐diamidino‐2‐phenylindole dihydrochloride (DAPI) and 0.1μM phalloidin‐atto‐550 in PBS for 75 min. Finally, the cells were washed with PBS containing 1% Tween, and washed twice with PBS afterward. Stained samples were mounted in a PDMS chamber filled with mowiol to ensure the MAC did not collapse.

### 4.9 Epifluorescence Microscopy

Fluorescence micrographs were obtained using a Leica DMi8 microscope using a Leica TL LED light source (P_max_ = 15W), a HC PL FLUOTAR L 40X 0.6 NA dry objective and a Hamamatsu‐C‐13440‐20C‐CL‐304422 camera set to 2x2 binning. Phase contrast images were taken with an exposure of 300ms, DAPI images with 200ms exposure and phalloidin images with 300ms exposure. The fluorescence illumination settings are listed in Table 1. Images were taken from a total of four replicas, divided over two biological replicas. Per condition, 13–19 images were taken.

**TABLE 1:** Illumination settings used for fluorescence microscopy.

Channel	Light source [nm]	Excitation filter [nm]	Dichroic mirror [nm]	Emission filter [nm]
DAPI	390	375–435	455	450–490
Phalloidin 550	555	540–580	585	592–668
CellTracker Green	470	450–490	500	500–550
Propidium Iodide	550	540–580	585	592–668

### 4.10 Cell Segmentation and Analysis from Fluorescence Microscopy

A *CellProfiler* pipeline to enhance image contrast, segment out cell nuclei and cytoskeleton, and compute morphological features of the identified shapes was built using the open source software CellProfiler (Figure S12B). The produced object outlines were manually reviewed and mis‐segmented cells due to a high degree of overlap or weak signal were excluded from further analysis. Cell and nucleus area, perimeter and eccentricity were calculated directly by CellProfiler. Using the image metadata, the computed cell area unit was converted from pixels to μm ^2^. Two derived cell shape parameters were calculated in addition to the parameters generated by CellProfiler: the ellipse form factor (perimeter/area ratio of the best fit ellipse) and the perimeter regularity (ratio of the CellProfiler‐generated form factor and the ellipse form factor).

### 4.11 Scanning Electron Microscopy Imaging and Analysis

MG‐63 cells cultured on MAC substrates were fixed using 3.7% formaldehyde solution in PBS for 20 min. Then, the fixed cells were rinsed with milliQ water twice, and dehydrated via washing with ethanol/water solutions of concentrations: 25%, 50%, 75%, 90%, and 99%. The dehydrated samples were dried through critical point drying (CPD) in a *Leica EM CPD‐300* using isopropanol as exchange fluid. The dried samples were sputter‐coated with a 5nm layer of gold before being imaged using a *Quanta 600* scanning electron microscope at high‐vacuum mode, 5.00kV and spot size 3.0. The images were analyzed using the open software ImageJ. The projected lengths of MAC bent by cells were measured, as well as the projected lengths of freestanding MAC. From the latter, the precise sample tilt was calculated. The vertical component of the projection line was corrected to in‐plane length using this sample tilt, and the horizontal component was calculated from the corrected vertical component and known length of the MAC.

### 4.12 Viability Testing After Magnetic Exposure / MAC Actuation

Three biological repeats were performed using five treatments: flat GelMA with no treatment (negative control), flat GelMA treated with a magnetic field, flat GelMA with triton x‐100 treatment (positive control), MAC with no magnetic field and MAC with magnetic field. All of these surfaces were sterilized in 70% ethanol for 15 min and washed with sterile PBS. Before cell seeding, the surfaces were incubated for 1 hour in cell culture medium at 37 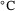, 5% ${\mathrm{CO}}_{2}$. MG‐63 cells were cultured to sub‐confluency before they were detached using Trypsin/EDTA. After centrifuging at 125g for 7 min, the trypsin solution was aspirated and replaced with 5mL of 0.5μg mL^−1^ CellTracker Green solution in serum‐free culture medium. The cells were resuspended and incubated for 20 min at 37 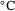, 5% ${\mathrm{CO}}_{2}$. Similarly, this solution was centrifuged, aspirated, and washed twice with 10mL of sterile PBS each. Finally, a similar washing step was done using 10mL of serum‐free culture medium. Next, the cells were centrifuged and transferred to normal culture medium and seeded on the prepared surfaces at a density of 5000/${\mathrm{cm}}^{2}$. The cells were left to adhere on the surfaces overnight, before their respective treatments. Before treatment, the cells were imaged for a base line cell count (CellTracker Green settings, Table 1). The treatment for the dead cell positive control consisted of 15 min incubation in 0.5% Triton X‐100 and washing once in PBS. After treatment, all samples were stained with 0.15mM propidium iodide (PI) for 5 min at 37 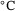, 5% ${\mathrm{CO}}_{2}$. A final imaging round was done after PI staining (CellTracker Green and Propidium Iodide settings, Table 1).

### 4.13 GFP‐YAP Transduction of HDFs and spy555 Staining

To generate normal Human Dermal Fibroblast cells stably expressing GFP‐YAP, lentivirus particles were generated in HEK293T LentiX cells, by polyethylenimine (PEI) transfection of the plasmids pLenti‐GFP‐YAP, pCMVR8.74, and pMD2.G. Medium was harvested three days after transfection, cleared by 500g centrifugation for 5 min. The supernatant was used to transduce nHDF cells that were selected for successful transduction with 0.5 μg mL^−1^ puromycin. pCMVR8.74 and pMD2.G were a gift from Didier Trono (RRID:Addgene_22036 and RRID:Addgene_12259). To generate pLenti GFP‐YAP, the GFP‐YAP construct was cloned from pEGFP‐C3‐hYap1 (a gift from Marius Sudol, RRID:Addgene_17843) into pENTR1A‐Jag1 [39] using Gibson Assembly and transferred to pLenti DEST‐PGK‐Puro (a gift from Eric Campeau and Paul Kaufman, RRID:Addgene_19068) using LR‐Clonase.

Spydna‐555 staining kit was purchased from Spirochrome AG, and diluted in phenol red‐free culture medium following the protocol on the Spirochrome product information (https://spirochrome.com/documents/202003/ datasheet_SPY555‐DNA_202003.pdf). Adhered transduced HDF cells were washed once with PBS before labeling them for 60 min at 37 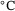, 5% ${\mathrm{CO}}_{2}$. After this, cells were washed again with PBS before adding normal culture medium to run the time‐lapse experiments.

### 4.14 Time‐Lapse Analysis

Spy555‐stained GFP‐YAP HDF cells were cultured at at 37 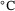, 5% ${\mathrm{CO}}_{2}$ for 4 h. For the actuated MAC device, the actuation settings were 120mT to both coils at 0.5Hz frequency using a triangle wave pattern. During this period, images were taken at 5 min intervals using a *CytoSmart* LUX3FL microscope (illumination settings in Table 2). The fluorescent images were processed using a *CellProfiler* pipeline (Figure S12D) to segment the nuclei regions and measure their YAP intensities. A custom Python script (Figure S12C) was used to track the segmented nuclei over time and provide a time‐dependent per‐object dataset.

**TABLE 2:** Illumination settings of the *CytoSmart* LUX3FL.

Channel	Excitation filter [nm]	Emission filter [nm]
GFP (green channel)	452/45nm	512/23nm
Spy555 (red channel)	561/14nm	630/90nm

### 4.15 Statistical Analysis

For all experiments, statistical analysis was carried out using *R Studio* software.

#### 4.15.1 MAC Deflection Assessment

A custom Python program was used to measure the tip displacement of N = 267 of the artificial cilia from bright field microscopic images. These measurements were used without further transformation or normalization for analysis. The mean and 95% confidence intervals for the MAC tip displacement values were calculated and plotted in Figure 1.

#### 4.15.2 MG‐63 Attachment and Viability Assessment

The experiment was performed for N = 3 biological replicates, with 5000–10.000 cells per replicate per experimental condition. Cell counts were performed by microscopy before and after treatment, and their ratios were calculated per experimental condition to yield cell attachment values. After treatment, the count of dead (PI‐stained) cells relative to the sample's total cell count was calculated to yield cell viability values. No further data transformation was applied. The means and standard deviations were calculated per replicate per experimental condition, and p‐values for the differences between individual experimental conditions were calculated using a two‐sided Student t‐test.

#### 4.15.3 MG‐63 Morphology Assessment

A *CellProfiler* pipeline was used to segment cells from fluorescent images and to calculate their area and eccentricity. The calculated cell area was converted to $\mu m^{2}$ using image metadata. Sample sizes range from 80–148 cells per condition. The area and eccentricity of the cells was displayed in box plots containing medians and quartile markers, excluding outliers. P‐values comparing each of the experimental conditions were calculated using ANOVA.

## Author Contributions

**Roel Kooi**: methodology, software, validation, formal analysis, investigation, data curation, writing – original draft, and visualization. **Tanveer Ul Islam**: methodology, visualization, writing – review and editing, and supervision. **Oscar Stassen**: methodology and writing – review and editing. **Naomie Amsing**: investigation and methodology. **Jan de Boer**: methodology, writing – review and editing, resources, and supervision. **Jaap den Toonder**: conceptualization, methodology, resources, writing – review and editing, supervision, project administration and funding acquisition.

## Conflicts of Interest

The authors declare no conflicts of interest.

## Supporting information


**Supporting File**: adhm70877‐sup‐0001‐SuppMat.pdf.

## Data Availability

The data supporting the findings of this study are available from the corresponding author upon reasonable request.
