# Supplementary material for: Controllable Dynamic Mechanical Cell Stimulation using Magnetically Actuated Artificial Cilia
Source: Adv Healthc Mater. 2026 Feb 1;15(18):e00001. doi: 10.1002/adhm.202600001 (PMC13176535; doi:10.1002/adhm.202600001)
Supplement: Supplementary file 1 — Supporting File: adhm70877‐sup‐0001‐SuppMat.pdf. [file ADHM-15-0-s001.pdf]

Supporting Information

Controllable Dynamic Mechanical Cell Stimulation using Magnetically Actuated Artificial Cilia

Roel Kooi[1,3]. Tanveer Ul Islam[1,3], Oscar M.J.A. Stassen[1,3], Naomie Amsing[1,3], Jan de Boer[2,3], and Jaap M.J. den Toonder[1,3,*]

1. Department of Mechanical Engineering, Eindhoven University of Technology, 5600MB Eindhoven, The Netherlands
2. Department of Biomedical Engineering, Eindhoven University of Technology, 5600MB Eindhoven, The Netherlands
3. Institute for Complex Molecular Systems, Eindhoven University of Technology 5600MB Eindhoven, The Netherlands

* Corresponding author: Jaap M.J. den Toonder, j.m.j.d.toonder@tue.nl

# MAC conn.	$\overline{MAC-}$[%]	$\overline{MAC+}$[%]	t	degr. of freedom	p-value
0	25.80038	13.21056	-1.4302	3.1405	0.2441
1	22.34614	21.78664	-0.20951	3.413	0.8459
2	18.78794	27.06755	1.6839	3.7351	0.1725
3	14.19010	17.72143	1.1495	2.0016	0.3692
4	7.931403	6.953443	-0.45098	3.9722	0.6755
5	6.102989	8.094429	0.52652	3.3013	0.6319

Table S1: Welch 2-sample t-tests for the MAC+ / MAC- comparisons of n_c-values.

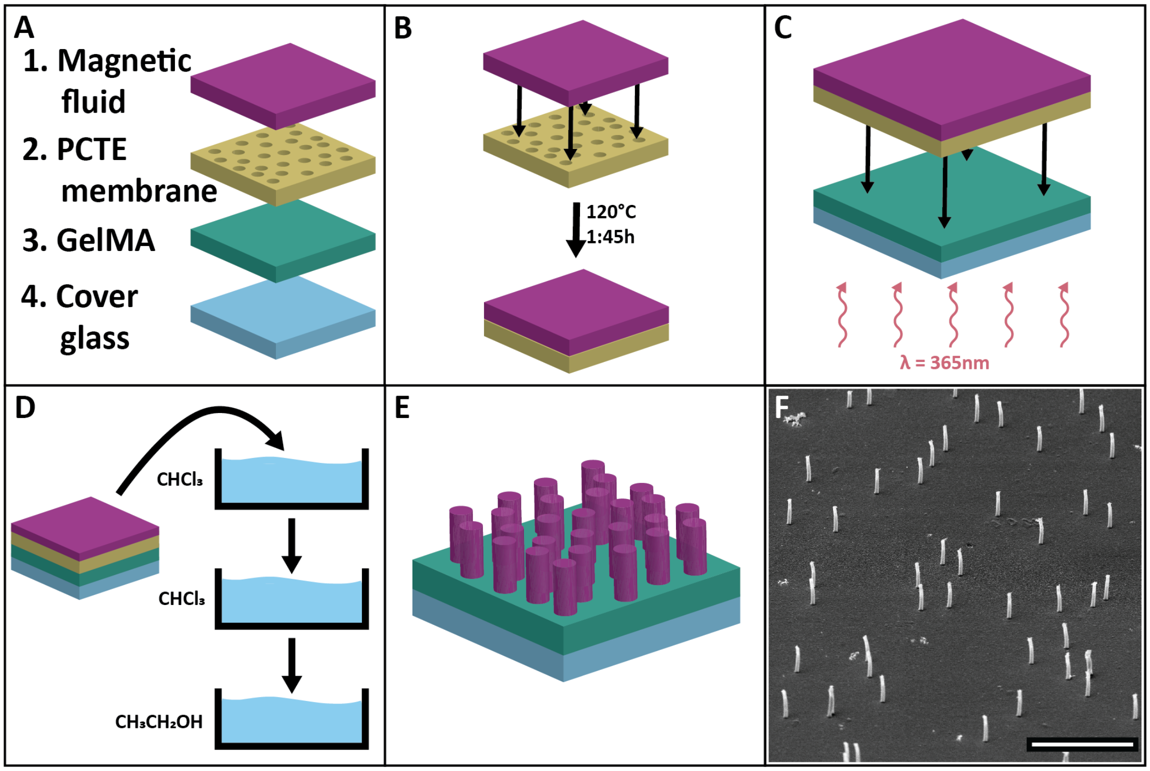

Figure S1: Detailed fabrication process of the MAC. **A:** Full layer-by-layer make-up of the MAC device. **B:** Step 1, applying magnetic fluid to the PCTE membrane, pushing it through the pores and leaving a layer of magnetic fluid on top before thermally curing the magnetic fluid. **C:** Step 2, pressing the cured artificial cilia inside the PCTE membrane onto a GelMA layer before curing the GelMA using UV irradiation, fusing the artificial cilia to the base layer. **D:** Step 3: washing the fused device using chloroform to dissolve the PCTE membrane, leaving (**E**) the final device. **F:** Scanning electron micrograph showing the final MAC device, taken on a 45° viewing angle. Artificial cilia diameter is 2μm and length is 23μm. Scale bar represents 50μm.

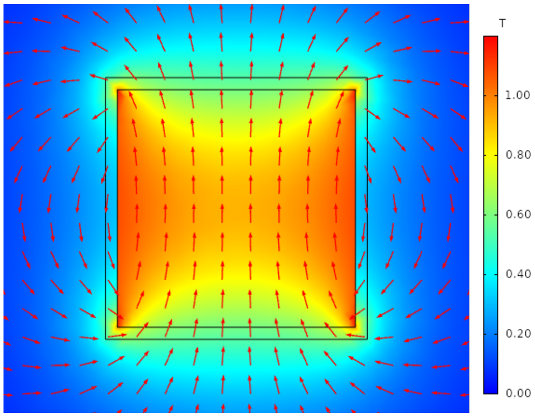

Figure S2: Simulated magnetic flux density (color plot) and direction (vector plot) for a neodymium cube magnet with side 5mm.

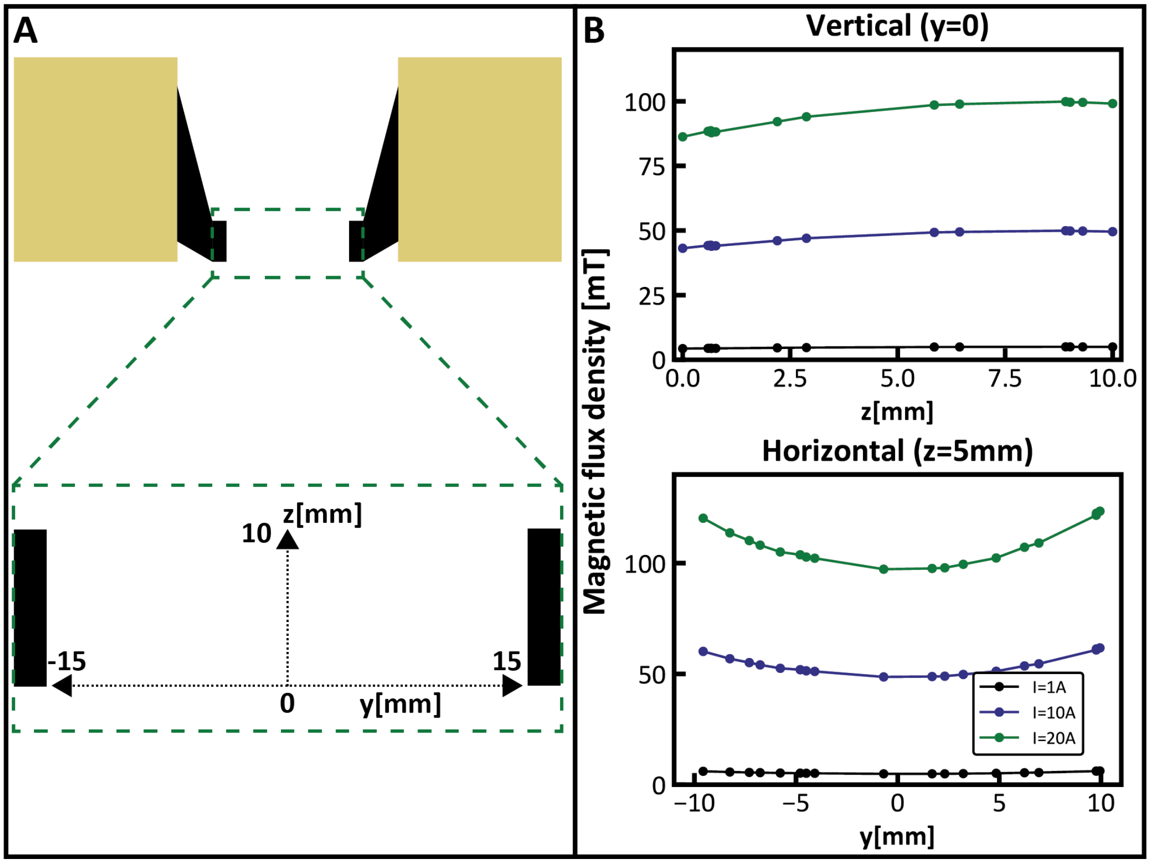

Figure S3: **A:** Schematic side view of the electromagnet, indicating the directions of the magnetic flux density plots. **B:** The simulated magnetic flux density, vertically exactly between the steel poles (y=0mm, top) and horizontally between the poles at half height (z=5mm, bottom).

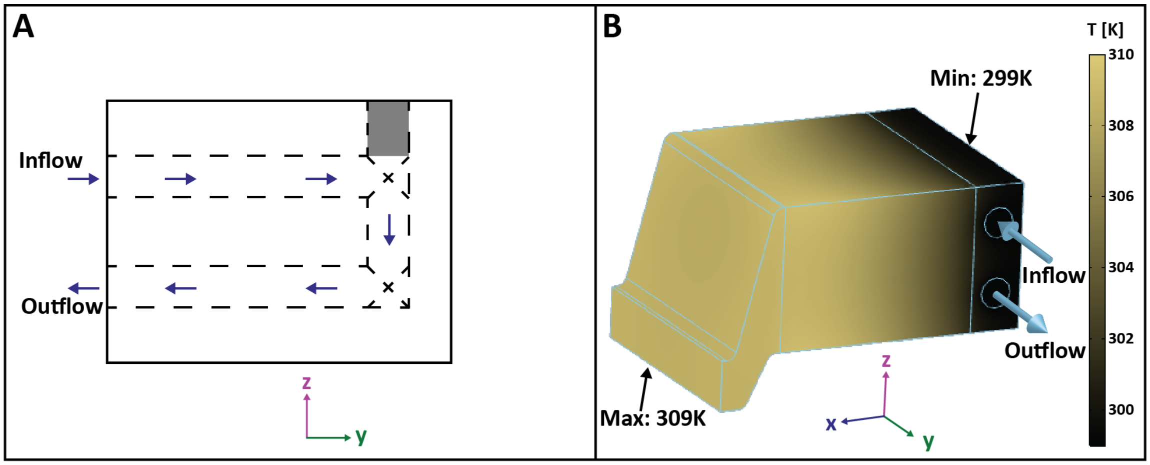

Figure S4: **A:** Wireframe drawing of the back of the steel core, showing the U-bend path for cooling water. The grayed-out area was drilled through and filled up afterwards to connect the two horizontal channels. **B:** Surface heat map plot of the temperature on the steel core, simulated in *COMSOL Multiphysics 5.5*, assuming maximum power (44W) and room temperature cooling water.

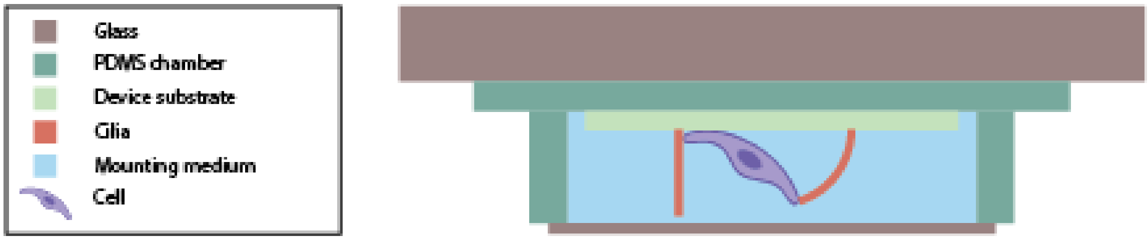

Figure S5: Schematic cross-section of the specialized mounting device used for fluorescence microscopy on AMS-based static MAC devices.

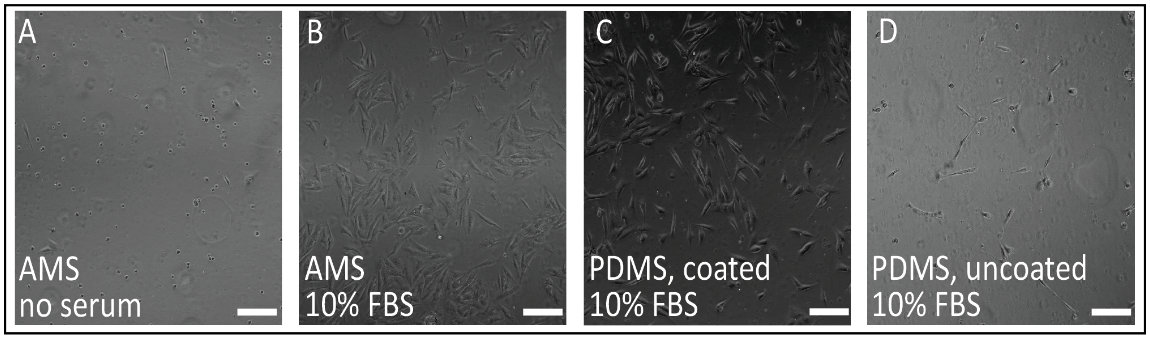

Figure S6: Bright field microscopic images of MG-63 cultured on flat surfaces of AMS (**A, B**) and PDMS (**C, D**). **A:** Flat AMS surface, cells cultured without FBS as a negative control for cell attachment. **B:** Flat AMS surface, cells cultured with 10% v/v FBS. **C:** Flat PDMS surface coated with 5μg/cm^2 of collagen-I. **D:** Flat PDMS surface, uncoated.

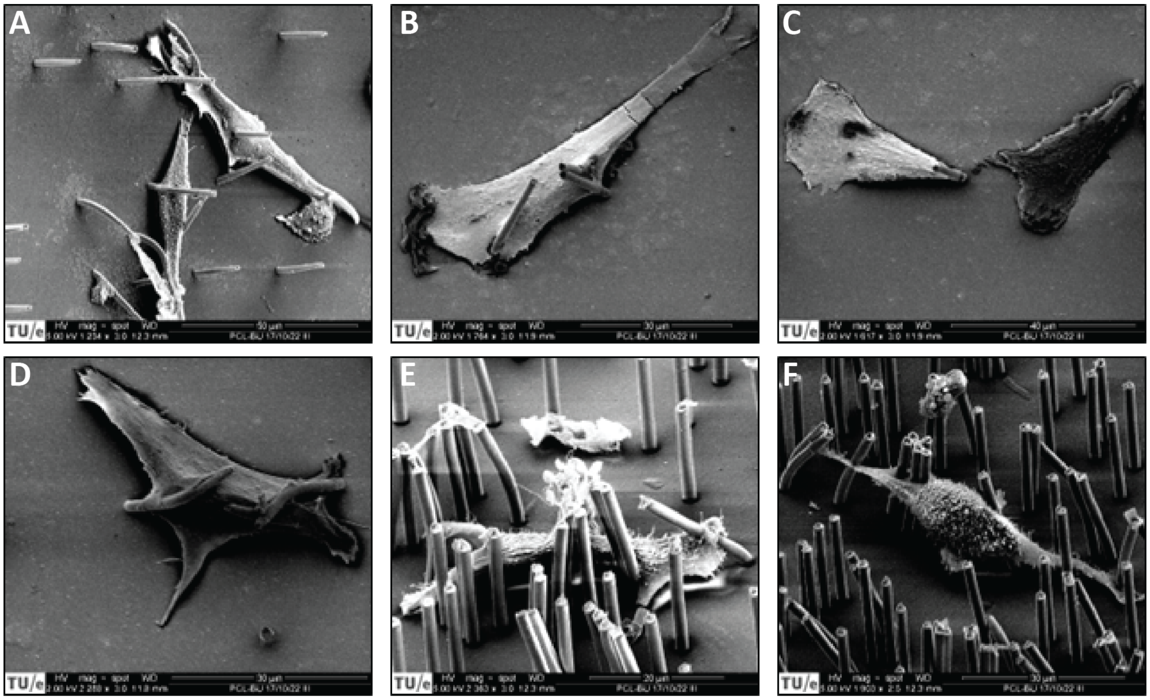

Figure S7: Additional SEM micrographs of MG-63 cells cultured on MAC devices. **A-D:** Areal density 100,000cm^{-2}. **E-F:** Areal density 1,000,000cm^{-2}.

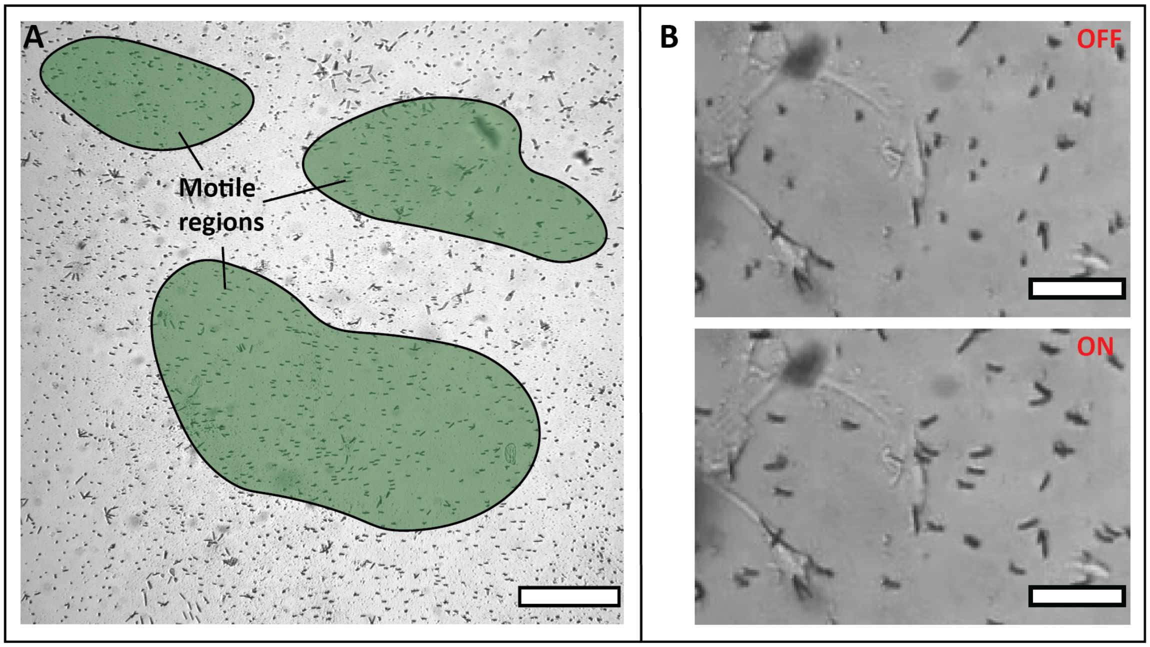

Figure S8: **A:** Brightfield image of a MAC device (top view), showing several regions with good cilia motility. Scale bar is 200μm. **B:** Brightfield images showing MG-63 cells on a MAC device, comparing the unactuated 'OFF'-state (top) to the 'ON'-state (bottom) where the magnet is switched on. Scale bars are 50μm.

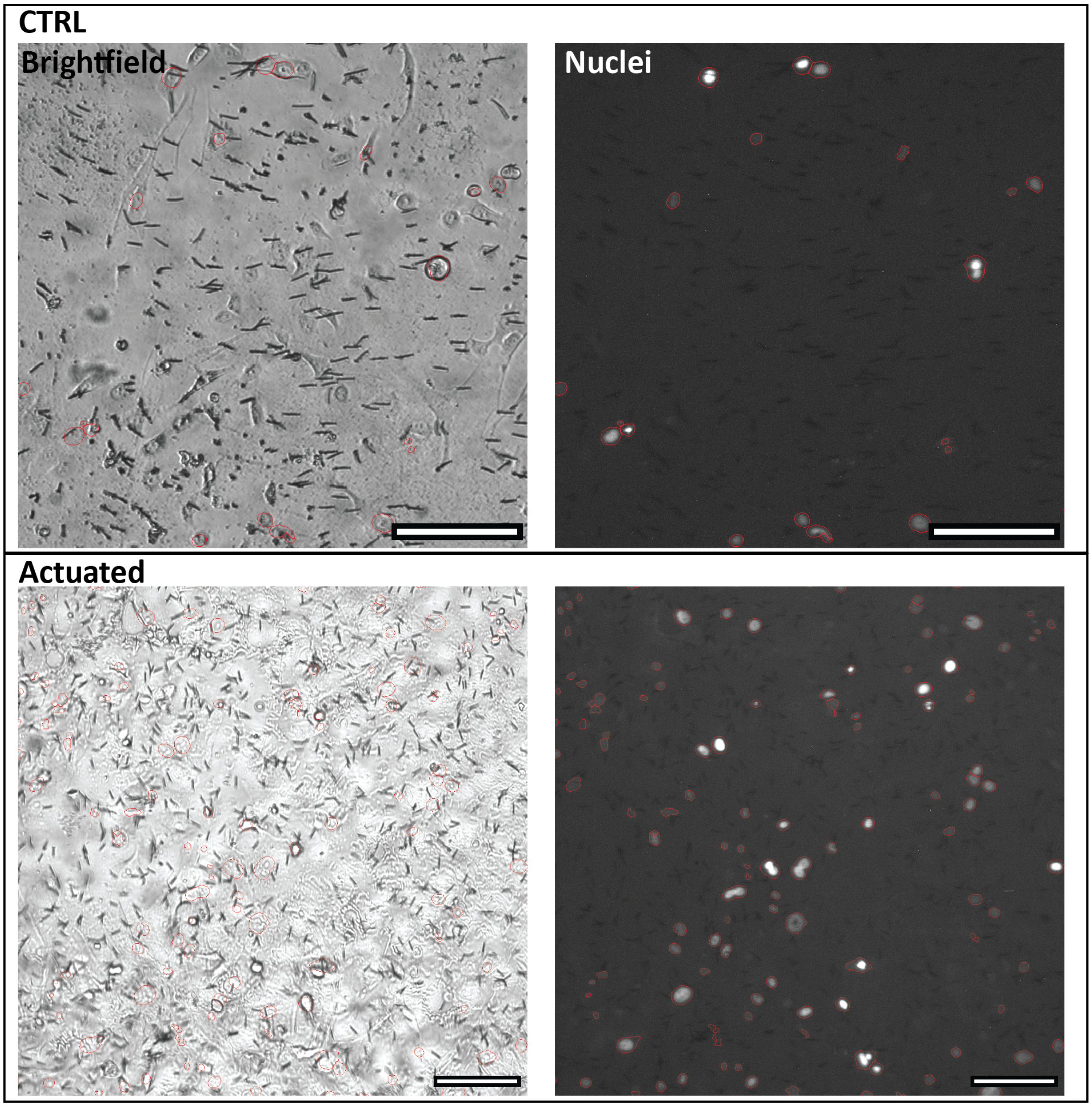

Figure S9: SPY555 nuclear staining quality of HDFs for static MAC (CTRL) and actuated MAC. Brightfield images (left) show more cells than have detectable SPY555 signal (right).

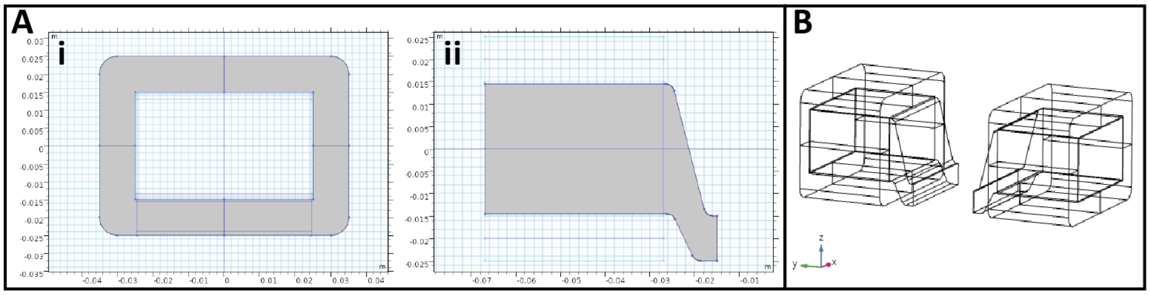

Figure S10: Geometry sketches of the model used to simulate electromagnetic field strength. **A:** Cross sections of the copper coil geometry (i) and the iron core geometry (ii). **B:** 3D wireframe ketch of the full model geometry.

CALCULATION TABLE

	Vial	Full	No solvent	Netto	[gr]
**Residue**	16.1374	39.997	x	23.8596	
**Sediment**	16.132	x	16	0.1665	
**Sample**	16.1872	16.6720	16.2183	Dry	0.0311
				Total	0.4848

LOSS TABLE

	Dirty	Clean	Loss	[gr]
**Bottle**	146.3634	146.3421	0.0213	
**Stirrer**	9.2761	9.2745	0.0016	
**Glass rod**	5.6721	5.6678	0.0043	
		Sum	0.0272	

**Theoretical yield**	0.858

**To be calculated**	**Definition**	**Value**	**Unit**
% Coated particles in RES	Dry weight sample/total weight sample	6.4150165	%
Weight coated particles in RES	% coated particles in RES x netto weight RES	1.5305973	gr
Total amount of polymer-coated pa	Sum of loss amount and RES	1.5577973	gr
Total amount of particles (no coati	Theoretical yield - particles in SED	0.6915	
Amount of particles in polymer	Total amount of particles/total amount of polymer	44.389601	%
**Dissolve solution to desired concentration**			
Desired weight percentage		40	%
Particle weight	Amount of particles in polymer x weight coated particles in RES	0.679426	gr
Target weight polymer (total weight	Particle weight/desired weight percentage	1.6985651	gr
Amount of AMS-mixture to add	Target weight polymer - weight coated particles in RES	1.019139	gr
**Ratio AMS:crosslinker = 10:1**			
Amount of AMS already present	Target weight polymer- particle weight	1.019139	gr
Amount of crosslinker to add	Grams of AMS to add/11	0.092649	gr
Amount of AMS base to add	Grams of AMS present - (weight coated particles in RES - particle weight)	0.0753188	gr

Figure S11: Calculation sheet used for tuning magnetic fluid concentrations. All percentages are % w/w.

A **B** **C** **D**

Figure S12: Links to GitHub projects of custom scripts used for data processing and/or analysis. **A:** Python code used for the measurement of cilia tip deflection. **B:** CellProfiler pipeline used to measure morphological features and f-actin intensity of MG-63 cells. **C:** Python code used to track nuclei objects over time. **D:** CellProfiler pipeline used to segment nuclei from CytoSmart LUX3FL images and measure YAP intensity.
